# Eplontersen for Hereditary Transthyretin Amyloidosis With Polyneuropathy: An Exploratory Analysis of Treatment Effect in Male and Female Patients

**DOI:** 10.1002/mus.70230

**Published:** 2026-04-06

**Authors:** Márcia Waddington Cruz, John L. Berk, Yeşim Parman, Morie Gertz, Sami Khella, Markus Weiler, T. Jesse Kwoh, Maksym Pola, Barry Reicher, Jonatan Nåtman, Noel R. Dasgupta, Jonas Wixner

**Affiliations:** ^1^ CEPARM Federal University of Rio de Janeiro Rio de Janeiro Brazil; ^2^ Amyloidosis Center Américas‐Samaritano‐Vitória Hospital Complex Rio de Janeiro Brazil; ^3^ Boston University School of Medicine Boston Massachusetts USA; ^4^ İstanbul Üniversitesi ‐ Istanbul Tıp Fakültesi Istanbul Turkey; ^5^ Department of Hematology Mayo Clinic Rochester Minnesota USA; ^6^ University of Pennsylvania School of Medicine Philadelphia Pennsylvania USA; ^7^ Amyloidosis Center and Department of Neurology Heidelberg University Hospital Heidelberg Germany; ^8^ Clinical Development Ionis Pharmaceuticals, Inc. Carlsbad California USA; ^9^ Late Cardiovascular, Renal, Metabolism BioPharmaceuticals R&D Gothenburg Sweden; ^10^ BioPharmaceuticals R&D Gaithersburg Maryland USA; ^11^ BioPharmaceuticals Business Unit Gothenburg Sweden; ^12^ Indiana University School of Medicine Indianapolis Indiana USA; ^13^ Amyloidosis Centre, Department of Public Health and Clinical Medicine Umeå University Umeå Sweden

**Keywords:** ATTRv‐PN, eplontersen, female, male, quality of life

## Abstract

**Introduction/Aims:**

Eplontersen is approved in multiple regions for adults with hereditary transthyretin amyloidosis with polyneuropathy (ATTRv‐PN). This exploratory analysis was conducted to evaluate the treatment effect of eplontersen by sex in patients with ATTRv‐PN from the NEURO‐TTRansform trial (NCT04136184).

**Methods:**

Participants in NEURO‐TTRansform who received ≥ 1 dose of eplontersen were included in this analysis; participants in the inotersen reference group who switched to eplontersen were excluded. Eplontersen was evaluated in the overall patient population and in a subgroup with cardiomyopathy. Change from baseline in the NEURO‐TTRansform primary endpoints of serum transthyretin (TTR) levels to Week 65, modified Neuropathy Impairment Score +7 (mNIS+7) composite score to Week 66, and Norfolk Quality of Life‐Diabetic Neuropathy (Norfolk QoL‐DN) total score to Week 66 were evaluated. A historical placebo group from the NEURO‐TTR trial (NCT01737398) was included as a control.

**Results:**

The eplontersen group comprised 100 (69.4%) male and 44 (30.6%) female patients. The placebo group comprised 41 (68.3%) male and 19 (31.7%) female patients. For both sexes, treatment with eplontersen decreased serum TTR levels by > 80% (vs. reductions of 3%–13% with placebo), maintained mNIS+7 scores, and improved Norfolk QoL‐DN scores (vs. deterioration with placebo). The treatment effect of eplontersen was similar in male and female patients with cardiomyopathy.

**Discussion:**

Eplontersen halted neuropathy impairment and improved quality of life versus placebo to a similar degree in both male and female patients with ATTRv‐PN, including those with cardiomyopathy. These findings support the use of eplontersen as an effective treatment for ATTRv‐PN regardless of sex.

## Graphical abstract

This exploratory analysis of data from the NEURO‐TTRansform trial evaluated sex‐specific responses in patients with ATTRv‐PN who had received eplontersen in NEURO‐TTRansform versus a historical placebo. Eplontersen can lead to substantial decreases in percentage serum TTR levels, arrest neuropathy impairment, and improve patient quality of life, regardless of patient sex.

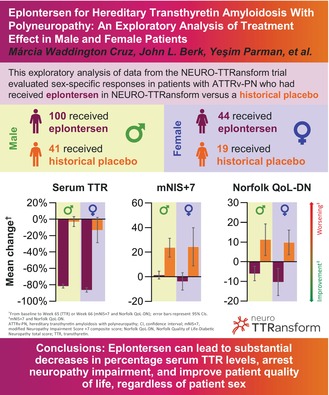

**Abbreviations**
ASO: antisense oligonucleotide
ATTR: transthyretin amyloidosis
ATTRv: hereditary transthyretin amyloidosis
ATTRv‐CM: hereditary transtheyretin amyloidosis with cardiomyopathy
ATTRv‐PN: hereditary transthyretin amyloidosis with polyneuropathy
CI: confidence interval
CM: cardiomyopathy
IQR: interquartile range
LSM: least squares mean
LSMD: least squares mean difference
mBMI: modified body mass index
mNIS+7: modified Neuropathy Impairment Score +7
Norfolk QoL‐DN: Norfolk Quality of Life‐Diabetic Neuropathy
NSC: Neuropathy Symptom and Change
PCS: Physical Component Summary
PND: Polyneuropathy Disability
QoL: quality of life
SD: standard deviation
SE: standard error
SF‐36: 36‐Item Short‐Form Health Survey
THAOS: Transthyretin Amyloidosis Outcomes Survey
TTR: transthyretin

## 1 Introduction

Hereditary transthyretin (ATTRv) amyloidosis is a rare, progressive, and fatal autosomal dominant disorder caused by *transthyretin* (*TTR*) gene alteration [1].

The global Transthyretin Amyloidosis Outcomes Survey (THAOS) registry observed that most symptomatic patients with transthyretin (ATTR) amyloidosis were male (70.8%) [2]. Sex differences in ATTRv amyloidosis with cardiomyopathy (ATTRv‐CM) have been observed; the disease is more prevalent in male patients, who are also typically younger at time of diagnosis than female patients [3]. Differences in disease presentation have also been noted between male and female patients with wild‐type ATTR amyloidosis (ATTRwt); it has been shown that female patients tend to present later, and with less severe cardiac impairment but more severe neurologic impairment, compared with male patients [4]. However, some analyses have suggested that the prevalence of ATTRwt‐CM in female patients may be underestimated due to sex‐related biases in identifying the condition [3, 5].

Less is known about sex differences in ATTRv amyloidosis with polyneuropathy (ATTRv‐PN). There is little data on the prevalence of ATTRv‐PN in males versus females, although one global epidemiological study of 542 individuals with ATTRv‐PN found that 69% were male [6]. Evidence from a single‐center study in patients with the *Val30Met* (*p.Val50Met*) variant suggests that male patients with ATTRv‐PN are less likely than female patients to respond to treatment with the TTR stabilizer tafamidis [7]. We hypothesized that there may also be a relationship between sex and ATTRv‐PN progression/outcomes following treatment with eplontersen. To date, differences in specific treatment effects in male and female patients are mostly unstudied, although one clinical study of inotersen reported no statistically significant differences between male and female patients with ATTRv‐PN in changes from baseline in mNIS+7 and Norfolk QoL‐DN scores over a 66‐week treatment period [8]. Randomized trials evaluating active treatment in patients with ATTRv amyloidosis have often been performed in predominantly male populations [8, 9, 10], therefore, data in female patients are lacking. Preclinical models provide evidence that sex hormones, including estrogen and testosterone, influence TTR levels [3, 11, 12, 13], suggesting that there may be physiological mechanisms underlying potential differential disease presentation, progression, and/or treatment response patterns in male and female patients. In addition, female patients may exhibit stronger immune responses than male patients [14, 15], which may play a role in modulating amyloidogenesis or mitigating disease progression.

Eplontersen is an antisense oligonucleotide (ASO) approved in multiple countries and regions, including the United States, Canada, and the European Union, for use in adults with ATTRv‐PN [16, 17, 18]. In the NEURO‐TTRansform Phase 3 trial, eplontersen significantly reduced serum TTR levels through 65 weeks, and arrested neuropathy impairment and improved quality of life (QoL) through 66 weeks, versus historical placebo in adult patients with ATTRv‐PN [19].

The objective of this exploratory analysis was to evaluate the treatment effect of eplontersen in male and female patients from the NEURO‐TTRansform trial, both in the overall patient population and in a subgroup of patients with ATTRv‐CM.

## 2 Methods

### 2.1 Trial Design

The open‐label, Phase 3 NEURO‐TTRansform clinical trial (NCT04136184) evaluated the efficacy and safety of subcutaneous eplontersen 45 mg every 4 weeks until Week 81 [19, 20]. The inclusion criteria and endpoints of NEURO‐TTRansform were designed to be comparable with those used in the NEURO‐TTR clinical trial (NCT01737398) to enable comparison with a prespecified historical placebo arm derived from that trial [8, 20]. Detailed methods and results of the NEURO‐TTRansform trial, including the switch analyses, have been published previously [8, 19, 20, 21, 22].

The trial protocol for each study was approved for each participating center by the relevant local institutional review boards or ethics committees [8, 20]. NEURO‐TTRansform was conducted in accordance with the International Council for Harmonisation guidelines, and written informed consent was provided by all patients before enrollment.

### 2.2 Patients

In the primary study, patients were adults with a documented genetic mutation in the *TTR* gene who had symptoms and signs consistent with ATTRv‐PN and Coutinho Stage 1 or 2 disease [19, 20].

All participants who were randomized to eplontersen and who received at least one dose of eplontersen in NEURO‐TTRansform could be included in this analysis.

The cardiomyopathy (CM) subgroup in this analysis was defined per the published NEURO‐TTRansform statistical analysis plan [19]. Male and female patients either had a diagnosis of ATTR‐CM at study entry, or they had baseline interventricular septum thickness ≥ 13 mm on echocardiogram with no history of hypertension or ventricular hypertrophy and no two consecutive systolic blood pressure readings of ≥ 150 mmHg during the study, with no adjustments made by sex.

### 2.3 Outcome Measures

This analysis evaluated the treatment effect of eplontersen on patients according to sex, both in the overall population and in a subgroup of patients with CM. As female patients with ATTRv‐CM tend to have lower interventricular septum and posterior wall thickness than male patients [23], a sensitivity analysis of the CM subgroup was performed in which the criterion used to define heart wall thickness was adjusted to ≥ 12 mm for female patients.

#### 2.3.1 NEURO‐TTRansform Primary Endpoints

Changes from baseline to Week 65 were measured for serum TTR (g/L) [19] and reported as mean percentage change.

Changes from baseline to Week 66 were measured for the modified Neuropathy Impairment Score +7 (mNIS+7) composite score (range, −22.3 to 346.3) and the Norfolk Quality of Life‐Diabetic Neuropathy (Norfolk QoL‐DN) total score (range, −4 to 136) [19]. Higher scores indicate worsening neuropathy and poorer QoL [19].

Percentage change from baseline to Week 65 in serum TTR levels, and changes from baseline to Week 66 in the mNIS+7 and Norfolk QoL‐DN scores were also measured in a subgroup of male and female patients with CM.

#### 2.3.2 NEURO‐TTRansform Secondary Endpoints

Changes from baseline to Week 65/66 were measured for the Neuropathy Symptom and Change (NSC) score, the Physical Component Summary (PCS) score of the 36‐Item Short‐Form Health Survey (SF‐36), the modified body mass index (mBMI), and the Polyneuropathy Disability (PND) scale. For the NSC, scores range from 0 to 114 for male patients and from 0 to 108 for female patients, with higher scores indicating worsening symptoms [19]. For the SF‐36 PCS, scores range from 0 to 100, with higher scores indicating improving physical health–related QoL [19]. The mBMI (BMI [kg/m^2^] × albumin [g/L]) measures nutritional status and is adjusted for low serum albumin levels compared with BMI, with higher scores indicating a better health state [24, 25]. On the PND scale, lower scores indicate more preserved function (Table S1) [26].

### 2.4 Statistical Analysis

These analyses included participants with both baseline and at least one post‐baseline value for each respective endpoint. Arithmetic means and 95% confidence intervals (CIs) were calculated for all continuous efficacy endpoints.

Post hoc statistical analyses for treatment effect of eplontersen versus placebo for male versus female patients were performed for the continuous efficacy endpoints in the overall population: serum TTR levels (percentage change from baseline); mNIS+7, Norfolk QoL‐DN, NCS, SF‐36 PCS, and mBMI (change from baseline). These analyses were based on a mixed‐effects model with repeated measures with fixed categorical effects for treatment, time, sex, treatment‐by‐time interaction, treatment‐by‐sex interaction, and treatment‐by‐time‐by‐sex interaction, and fixed covariates for the baseline value of the endpoint, with data up to Week 65 included. All endpoints were reported as least squares means (LSMs) with standard errors (SEs) and 95% CIs for changes from baseline, and LSM differences (LSMDs) with SEs and 95% CIs for comparisons of eplontersen with historical placebo within sex subgroups. P values for the treatment‐by‐sex interaction term at the Week 65/66 visit were obtained from joint tests based on the mixed‐effects model, using *R* v4.3.2 for analysis and the *nlm* package to estimate the linear mixed models, with a significance level of 5%. Analyses of the PND scale and of efficacy endpoints in the CM subgroup were descriptive with no formal statistical tests performed.

## 3 Results

### 3.1 Patients

Of 144 patients treated with eplontersen, 100 (69.4%) were male and 44 (30.6%) were female. The historical placebo group (*n* = 60) comprised 41 (68.3%) male and 19 (31.7%) female patients. In general, male patients were younger, had a longer duration of disease from onset of ATTRv‐PN symptoms, and a higher prevalence of CM at baseline than female patients receiving the same treatment (Tables 1 and 2). In the eplontersen group, the *Val30Met* variant with early onset disease (< 50 years old) was more common in male versus female patients, whereas the presence of the *Val30Met* variant with late‐onset disease (≥ 50 years old) was similar between male and female patients. Cardiac predominance (*Thr60Ala* [*p.Thr80Ala*] and *Val122Ile* [*p.Val142lle*] variants) was seen in only four male and four female patients treated with eplontersen. Approximately 20% of male and female patients treated with eplontersen were characterized as having a mixed phenotype based on their genotype (Table 2).

**TABLE 1:** Baseline demographics according to sex.

Characteristic	Male		Female	
	Eplontersen (*n* = 100)	Historical placebo (*n* = 41)	Eplontersen (*n* = 44)	Historical placebo (*n* = 19)
Mean (SD) age, years	51.7 (15.0)	59.2 (14.1)	56.1 (14.7)	60.3 (14.3)
Race, *n* (%)				
Asian	14 (14.1)	1 (2.4)	8 (18.2)	2 (10.5)
Black or African American	4 (4.0)	0	1 (2.3)	1 (5.3)
White	79 (79.8)	38 (92.7)	33 (75.0)	15 (78.9)
Other or multiple	2 (2.0)	2 (4.9)	2 (4.5)	1 (5.3)
Missing	1 (1.0)	0	0	0
Geographic region, *n* (%)				
North America	10 (10.0)	18 (43.9)	11 (25.0)	8 (42.1)
Central and Southern America	31 (31.0)	7 (17.1)	15 (34.1)	4 (21.1)
Europe, Australia, New Zealand	46 (46.0)	16 (39.0)	10 (22.7)	7 (36.8)
Taiwan	13 (13.0)	0	8 (18.2)	0
Mean (SD) body weight, kg	*n* = 100 72.6 (15.0)	*n* = 41 75.7 (18.0)	*n* = 41 64.8 (16.4)	*n* = 19 61.1 (14.3)
Mean (SD) BMI, kg/m^2^	*n* = 98 24.0 (4.5)	*n* = 41 24.5 (4.9)	*n* = 40 25.1 (5.8)	*n* = 19 23.5 (4.8)
Mean (SD) albumin, g/L	42.7 (2.9)	43.5 (3.0)	41.1 (2.7)	43.3 (3.2)

Abbreviations: BMI, body mass index; SD, standard deviation.

**TABLE 2:** Baseline clinical characteristics according to sex.

Characteristic	Male		Female	
	Eplontersen (*n* = 100)	Historical placebo (*n* = 41)	Eplontersen (*n* = 44)	Historical placebo (*n* = 19)
*TTR* variant, *n* (%)				
*Val30Met* early onset (< 50 years)	41 (41.0)	11 (26.8)	13 (29.5)	5 (26.3)
*Val30Met* late‐onset (≥ 50 years)	22 (22.0)	14 (34.1)	9 (20.5)	3 (15.8)
*Ala97Ser*	13 (13.0)	0	8 (18.2)	1 (5.3)
Cardiac predominance (*Thr60Ala* and *Val122Ile*)	4 (4.0)	7 (17.1)	4 (9.1)	2 (10.5)
Mixed, all others	20 (20.0)	9 (22.0)	10 (22.7)	8 (42.1)
Median (IQR) duration of disease from onset of ATTRv‐PN symptoms, months	*n* = 99 57.0 (37.5, 98.5)	*n* = 41 48.0 (24.0, 86.0)	*n* = 44 50.0 (27.8, 78.0)	*n* = 19 47.0 (35.0, 88.5)
Median (IQR) duration of disease from ATTRv‐PN diagnosis, months	33.0 (9.0, 63.8)	24.0 (9.0, 53.0)	25.5 (6.8, 45.0)	32.0 (6.5, 59.5)
Median (IQR) duration of disease from onset of ATTRv‐CM symptoms, months	*n* = 21 19.0 (10.0, 39.0)	*n* = 13 27.0 (7.0, 35.0)	*n* = 9 13.0 (13.0, 30.0)	*n* = 5 37.0 (29.0, 68.0)
Median (IQR) duration of disease from ATTRv‐CM diagnosis, months	*n* = 29 14.0 (4.0, 32.0)	*n* = 16 15.0 (5.5, 27.0)	*n* = 10 6.0 (1.2, 10.2)	*n* = 6 18.5 (4.2, 35.0)
Mean (SD) TTR level, g/L	*n* = 98 0.24 (0.07)	*n* = 41 0.15 (0.04)	*n* = 43 0.21 (0.08)	*n* = 18 0.15 (0.04)
Mean (SD) mNIS+7 composite score	83.2 (44.0)	78.5 (39.9)	76.9 (42.1)	66.6 (36.7)
Mean (SD) Norfolk QoL‐DN total score	*n* = 98 44.7 (25.6)	*n* = 40 49.3 (26.6)	*n* = 39 42.5 (29.3)	*n* = 19 47.4 (27.8)
Mean (SD) NCS total score	24.5 (12.9)	24.0 (12.6)	20.1 (10.7)	20.8 (12.7)
Mean (SD) SF‐36 PCS score	39.7 (9.0)	36.1 (8.6)	39.6 (9.9)	39.7 (12.0)
Mean (SD) mBMI	*n* = 98 1021.3 (220.3)	*n* = 41 1066.4 (238.3)	*n* = 40 1036.7 (270.8)	*n* = 19 1014.2 (207.2)
ATTRv‐PN stage, *n* (%)				
Stage 1	80 (80.0)	30 (73.2)	35 (79.5)	12 (63.2)
Stage 2	20 (20.0)	11 (26.8)	9 (20.5)	7 (36.8)
PND score, *n* (%)				
0	0	0	0	0
I	34 (34.0)	14 (34.1)	22 (50.0)	9 (47.4)
II	48 (48.0)	16 (39.0)	13 (29.5)	3 (15.8)
IIIa	12 (12.0)	9 (22.0)	4 (9.1)	6 (31.6)
IIIb	5 (5.0)	2 (4.9)	5 (11.4)	1 (5.3)
Missing	1 (1.0)	0	0	0
Previous treatment with tafamidis or diflunisal, *n* (%)	72 (72.0)	24 (58.5)	28 (63.6)	12 (63.2)
ATTRv‐CM at baseline, *n* (%)	29 (29.0)	16 (39.0)	10 (22.7)	6 (31.6)
CM subgroup, *n* (%)	36 (36.0)	22 (53.7)	13 (29.5)	8 (42.1)
CM subgroup, sensitivity analysis, *n* (%)	36 (36.0)	22 (53.7)	14 (31.8)	8 (42.1)

Abbreviations: ATTRv‐CM, hereditary transthyretin amyloidosis with cardiomyopathy; ATTRv‐PN, hereditary transthyretin amyloidosis with polyneuropathy; IQR, interquartile range; mBMI, modified body mass index; mNIS+7, modified Neuropathy Impairment Score +7; NCS, Neuropathy Symptom and Change; Norfolk QoL‐DN, Norfolk Quality of Life‐Diabetic Neuropathy; PCS, Physical Component Summary; PND, Polyneuropathy Disability; SD, standard deviation; SF‐36, Short Form‐36 questionnaire; TTR, transthyretin.

Baseline serum TTR levels were similar between male and female patients receiving placebo and slightly higher in male than female patients receiving eplontersen. Across both the eplontersen and historical placebo groups, most patients had PND score I or II impairment at baseline, with a greater proportion of male versus female patients classified as experiencing score II (Table 2).

In both the eplontersen and placebo groups, a higher proportion of male than female patients met the protocol‐defined criteria for CM. In the sensitivity analysis, one more female patient in the eplontersen group met the criteria for heart wall thickening compared with the overall analysis (Table 2).

### 3.2 Serum TTR Levels

**FIGURE 1:**
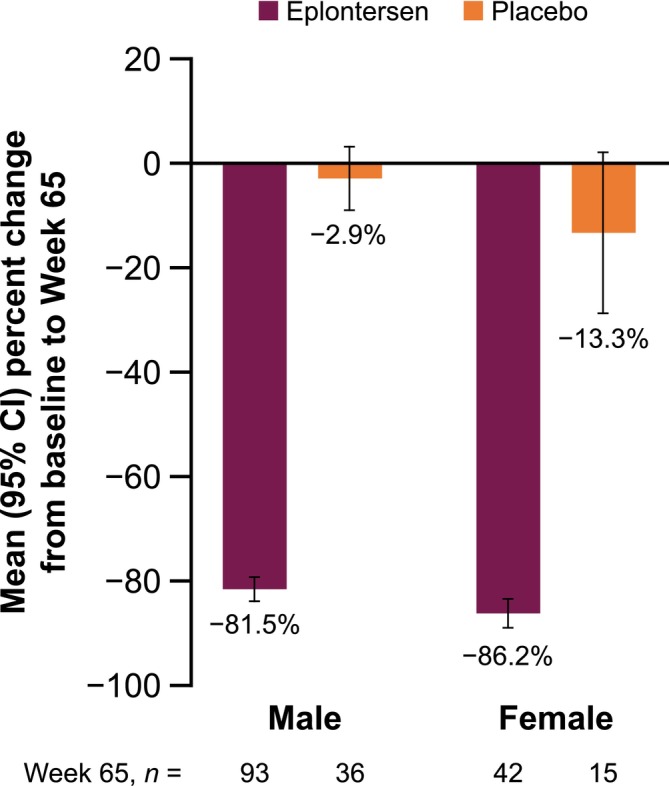
Percentage change from baseline to Week 65 in serum TTR levels in male and female patients. CI, confidence interval; TTR, transthyretin.

In both male and female patients, serum TTR levels decreased by > 80% at Week 65 in eplontersen patients compared with baseline levels. Among those randomized to placebo, serum TTR levels decreased by approximately 3% in male and approximately 13% in female patients from baseline levels (Figure 1).

LSMDs (95% CI) in percentage change in serum TTR from baseline to Week 65 for eplontersen compared with placebo were similar for male and female patients (Table S2).

In the CM subgroup, serum TTR levels showed a similar trend. Decreases of > 80% at Week 65 were seen in male and female eplontersen patients, whereas decreases of < 20% were seen in male and female placebo patients (Figure S1).

### 3.3 Neuropathy Impairment and QoL

**FIGURE 2:**
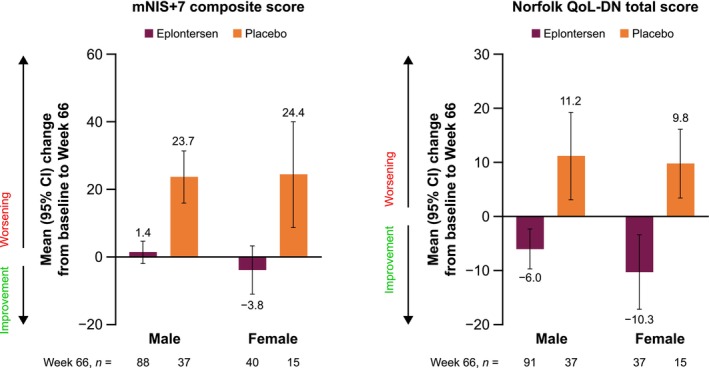
Change from baseline to Week 66 in mNIS+7 composite score and Norfolk QoL‐DN total score in male and female patients. CI, confidence interval; mNIS+7, modified Neuropathy Impairment Score +7; Norfolk QoL‐DN, Norfolk Quality of Life‐Diabetic Neuropathy.

In both male and female subgroups, mNIS+7 composite scores were maintained in eplontersen patients from baseline to Week 66, whereas a worsening of neuropathy impairment was observed in placebo patients. In both male and female subgroups, Norfolk QoL‐DN total scores improved in eplontersen patients from baseline to Week 66, whereas a worsening of QoL was observed in placebo patients (Figure 2). There were no statistically significant differences in the treatment effects of eplontersen versus placebo for male versus female patients (Table S2).

For both outcomes, mean changes from baseline to Week 66 in male and female patients in the CM subgroup were generally similar to those of the larger groups (Figure S2).

### 3.4 NCS, SF‐36 PCS, and mBMI

**FIGURE 3:**
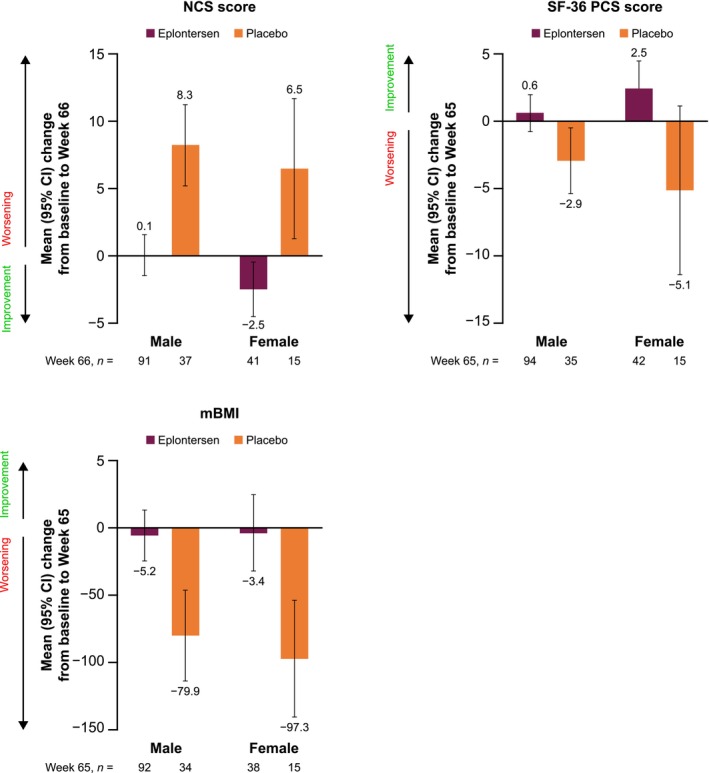
Change from baseline to Week 65/66 in NCS, SF‐36 PCS, and mBMI in male and female patients. CI, confidence interval; mBMI, modified body mass index; NCS, Neuropathy Symptom and Change; PCS, Physical Component Summary; SF‐36, Short Form‐36 questionnaire.

In both male and female patients, mean changes from baseline to Week 65 or 66 in the NCS and SF‐36 PCS scores were in favor of eplontersen compared with placebo (Figure 3). There were no statistically significant differences in the treatment effects of eplontersen versus placebo for male versus female patients (Table S2).

Mean changes from baseline to Week 65 in mBMI showed a similar trend in favor of eplontersen compared with placebo, for both male and female patients. Across both sexes, eplontersen patients showed weight maintenance, whereas weight decrease was seen in placebo patients (Figure 3). There was no statistically significant difference in the treatment effect of eplontersen versus placebo for male versus female patients (Table S2).

### 3.5 PND Score

**FIGURE 4:**
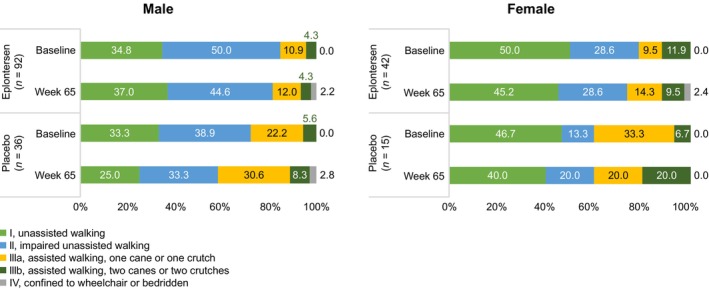
PND score distribution at baseline and at Week 65 in male and female patients. Percentages for patients with both baseline and Week 65 values. PND, Polyneuropathy Disability.

The percentage of male and female patients with PND scores I–IV at baseline and at Week 65 is shown in Figure 4.

Male patients receiving eplontersen maintained a less severe disease stage compared with those who received placebo, with a similar proportion of patients reporting an impairment score of I at both baseline measurement and Week 65. Over this same time interval, male patients in the placebo group demonstrated a shift in the distribution of PND scores, with a lower proportion with score I impairment and a greater proportion reporting an impairment score of IIIa, IIIb, and IV at Week 65.

Female patients receiving eplontersen reported similar impairment across categories at baseline and Week 65. Those receiving placebo, however, reported a substantially higher rate of score IIIb impairment at Week 65 compared with baseline (Figure 4).

## 4 Discussion

In this exploratory analysis of the NEURO‐TTRansform Phase 3 trial, eplontersen led to reductions in serum TTR levels (> 80%) and halted neuropathy progression (mNIS+7 composite score) compared with historical placebo, with consistent effects across sexes, indicating robust efficacy. Improvements in QoL with eplontersen, as measured by Norfolk QoL‐DN, NCS, and SF‐36 PCS scores, supported findings from mNIS+7 and were observed in both male and female patients. Eplontersen also preserved nutritional status (mBMI) and maintained stable PND scores, whereas historical placebo was associated with deterioration. Similar trends in serum TTR, mNIS+7 composite score, and Norfolk QoL‐DN total score were seen in the CM subgroup. Overall, these results indicate that eplontersen provides broad and consistent benefits in ATTRv‐PN, supporting its potential as a treatment option irrespective of patient sex.

Data on the differences in pathophysiology of ATTR‐PN between the sexes are limited. The NEURO‐TTR clinical trial of inotersen for ATTRv‐PN found that male and female patients receiving inotersen had statistically similar LSM changes from baseline to Week 66 in both mNIS+7 and Norfolk QoL‐DN [8]. An analysis from the THAOS registry noted that myocardial involvement may be more frequent and pronounced in male patients with ATTRv‐CM [27]. Sensory abnormalities were slightly more common in female patients, whereas autonomic impairment was more common in male patients [27]. Different frequencies of variant mutations were also observed in male and female patients [27]. In unselected populations, males typically report higher levels of QoL than females, which may be driven by socioeconomic and demographic factors [28, 29, 30]. In the current analysis, the Norfolk QoL‐DN total scores were numerically lower (indicative of better health state) at baseline in female patients compared with male patients, which might explain the shorter duration of symptoms reported by female patients, and the higher rate of female patients reporting a PND score of I (approximately 50% with eplontersen vs. 34% for male patients).

As shown in the current analysis, male patients may experience symptomatic disease earlier and with greater disease severity than female patients. Possible explanations for these findings include the cardioprotective effects of estrogen in women, which may reduce the impact of amyloid fibril deposition [3], the different impacts of estrogen and testosterone on cardiovascular physiology [3], and the contribution of low testosterone levels to increased oxidative stress and inflammation, and therefore accelerated vascular aging [31]. There may also be a role for genetic factors, with certain mutations associated with more severe disease [3, 32]. Despite these possible inherent sex differences, eplontersen provided benefits to patients regardless of sex.

The limitations of the current analysis include that, for ethical reasons, the NEURO‐TTRansform trial employed a historical placebo group from a previously conducted study. The use of a historical placebo means that NEURO‐TTRansform was effectively a single‐group active treatment study in terms of investigator and patient blinding, potentially leading to bias in outcome assessment, particularly for subjective endpoints. Further, the sex ratio was not uniform across all geographic regions, and differences in medical and care practices between geographic regions may affect results. The statistical analysis of the continuous efficacy endpoints requires validation through a new, independent study with a prespecified, confirmatory analysis plan in order to be considered definitive. The criteria for CM subgroup inclusion did not take into account differences in cardiac presentation between male and female patients, notably that female patients with ATTRv‐CM tend to have lower interventricular septum and posterior wall thickness than male patients [23]; however, a sensitivity analysis of the CM subgroup that adjusted the heart wall thickness criterion from ≥ 13 mm to ≥ 12 mm for female patients identified only one additional female patient. Finally, limited sample sizes precluded further analysis of these data.

## 5 Conclusion

These findings support the use of eplontersen as an effective treatment for ATTRv‐PN in both male and female patients. In addition, male and female patients with ATTRv‐PN with CM were found to benefit from eplontersen. Clinicians should consider eplontersen for all eligible patients, given the consistent efficacy observed across sexes and disease presentations.

## Author Contributions

**Márcia Waddington Cruz:** investigation, writing – original draft, writing – review and editing. **John L. Berk:** conceptualization, investigation, writing – original draft, writing – review and editing. **Yeşim Parman:** investigation, writing – original draft, writing – review and editing. **Morie Gertz:** investigation, writing – original draft, writing – review and editing. **Sami Khella:** investigation, writing – original draft, writing – review and editing. **Markus Weiler:** investigation, resources, writing – original draft, writing – review and editing. **T. Jesse Kwoh:** writing – original draft, writing – review and editing. **Maksym Pola:** conceptualization, methodology, writing – original draft, writing – review and editing. **Barry Reicher:** conceptualization, methodology, writing – original draft, writing – review and editing. **Jonatan Nåtman:** formal analysis, writing – original draft, writing – review and editing. **Noel R. Dasgupta:** investigation, resources, writing – original draft, writing – review and editing. **Jonas Wixner:** investigation, resources, writing – original draft, writing – review and editing.

## Funding

The NEURO‐TTRansform trial was sponsored by Ionis Pharmaceuticals Inc. The analysis presented here was supported by AstraZeneca.

## Conflicts of Interest

M.W.C. is a principal investigator for the NEURO‐TTRansform trial and is a consultant for Ionis. J.L.B. has participated in an Ionis and BridgeBio *ad hoc* advisory committee and is a consultant to Intellia and Alnylam. Y.P. has received honoraria from Pfizer, Alnylam, and Ionis. M.G. has received personal fees from Ionis, Alnylam, Prothena, Janssen, Sanofi, Juno, Physicians Education Resource, Johnson & Johnson, Celgene, and Research to Practice; served on the data and safety monitoring board for AbbVie; received research funding from Aptitude Health; received meeting fees from Ashfield and Sorrento; and developed educational materials for i3Health. S.K. is a consultant to Ionis, Pfizer, AstraZeneca, BridgeBio, and Alnylam. M.W. is a consultant to Akcea, Alnylam, Biogen, F. Hoffmann‐La Roche, Novartis, Novo Nordisk, Pfizer, Purpose Pharma, and Swedish Orphan Biovitrum AB; received speaker fees from Akcea, Alnylam, AstraZeneca, and Biogen; and received financial support for conference attendance from Akcea, Alnylam, AstraZeneca, BridgeBio, Ionis, and Pfizer; and is a member of the European Reference Network for Neuromuscular Diseases (ERN EURO‐NMD). T.J.K. was an employee and shareholder of Ionis during the conduct of the studies and is presently a contractor and shareholder of Ionis. M.P., B.R., and J.N. are employees of, and hold stock in, AstraZeneca. N.R.D. has been a consultant for Novo Nordisk, Eidos, Alnylam, AstraZeneca, BridgeBio, and Intellia/Regeneron; non‐financial writing assistance from Eidos; non‐financial travel support from the American Society of Hematology; and research funding from Ionis. J.W. is a consultant to Akcea, AstraZeneca, Alnylam, Pfizer, BridgeBio, and Intellia.

## Supporting information


**Table S1.** Polyneuropathy Disability disease staging.
**Table S2.** Treatment effect of eplontersen vs. placebo at Week 65/66 for male vs. female patients.
**Figure S1.** Percentage change from baseline to Week 65 in serum TTR levels in male and female patients with CM.
**Figure S2.** Change from baseline to Week 66 in mNIS+7 composite score and Norfolk QoL‐DN total score in male and female patients with CM.

## Data Availability

Data requests from qualified researchers will be considered once all three of the following criteria are met: (1) 12 months from marketing approval of the study drug in both the United States and European Union; (2) 18 months from conclusion of the study; and (3) 6 months from the publication of this article. For additional information, visit https://vivli.org/ourmembers/ionis.

## References

[1] D. Adams , V. Algalarrondo , M. Polydefkis , N. Sarswat , M. S. Slama , and J. Nativi‐Nicolau , “Expert Opinion on Monitoring Symptomatic Hereditary Transthyretin‐Mediated Amyloidosis and Assessment of Disease Progression,” Orphanet Journal of Rare Diseases 16, no. 1 (2021): 411. 34602081 10.1186/s13023-021-01960-9 PMC8489116

[2] L. Gentile , T. Coelho , A. Dispenzeri , et al., “A 15‐Year Consolidated Overview of Data in Over 6000 Patients From the Transthyretin Amyloidosis Outcomes Survey (THAOS),” Orphanet Journal of Rare Diseases 18, no. 1 (2023): 350. 37946256 10.1186/s13023-023-02962-5 PMC10636983

[3] A. Aimo , G. Panichella , M. Garofalo , et al., “Sex Differences in Transthyretin Cardiac Amyloidosis,” Heart Failure Reviews 29, no. 2 (2024): 321–330. 37566193 10.1007/s10741-023-10339-w PMC10942898

[4] C. M. Campbell , S. LoRusso , A. Dispenzieri , et al., “Sex Differences in Wild‐Type Transthyretin Amyloidosis: An Analysis From the Transthyretin Amyloidosis Outcomes Survey (THAOS),” Cardiol Ther 11, no. 3 (2022): 393–405. 35583798 10.1007/s40119-022-00265-7 PMC9381661

[5] R. K. Patel , A. Ioannou , Y. Razvi , et al., “Sex Differences Among Patients With Transthyretin Amyloid Cardiomyopathy ‐ From Diagnosis to Prognosis,” European Journal of Heart Failure 24, no. 12 (2022): 2355–2363. 36575133 10.1002/ejhf.2646 PMC10087683

[6] M. Waddington‐Cruz , H. Schmidt , M. F. Botteman , et al., “Epidemiological and Clinical Characteristics of Symptomatic Hereditary Transthyretin Amyloid Polyneuropathy: A Global Case Series,” Orphanet Journal of Rare Diseases 14, no. 1 (2019): 34. 30736835 10.1186/s13023-019-1000-1 PMC6368811

[7] C. Monteiro , J. S. Mesgazardeh , J. Anselmo , et al., “Predictive Model of Response to Tafamidis in Hereditary ATTR Polyneuropathy,” JCI Insight 4, no. 12 (2019): e126526. 31217346 10.1172/jci.insight.126526 PMC6629131

[8] M. D. Benson , M. Waddington‐Cruz , J. L. Berk , et al., “Inotersen Treatment for Patients With Hereditary Transthyretin Amyloidosis,” New England Journal of Medicine 379, no. 1 (2018): 22–31. 29972757 10.1056/NEJMoa1716793 PMC12611561

[9] D. Adams , I. L. Tournev , M. S. Taylor , et al., “Efficacy and Safety of Vutrisiran for Patients With Hereditary Transthyretin‐Mediated Amyloidosis With Polyneuropathy: A Randomized Clinical Trial,” Amyloid 30, no. 1 (2023): 1–9. 35875890 10.1080/13506129.2022.2091985

[10] M. S. Maurer , P. Kale , M. Fontana , et al., “Patisiran Treatment in Patients With Transthyretin Cardiac Amyloidosis,” New England Journal of Medicine 389, no. 17 (2023): 1553–1565. 37888916 10.1056/NEJMoa2300757 PMC10757426

[11] T. Quintela , C. H. Alves , I. Gonçalves , G. Baltazar , M. J. Saraiva , and C. R. A. Santos , “5Alpha‐Dihydrotestosterone Up‐Regulates Transthyretin Levels in Mice and Rat Choroid Plexus via an Androgen Receptor Independent Pathway,” Brain Research 1229 (2008): 18–26. 18634756 10.1016/j.brainres.2008.06.095

[12] T. Quintela , I. Gonçalves , G. Baltazar , C. H. Alves , M. J. Saraiva , and C. R. A. Santos , “17Beta‐Estradiol Induces Transthyretin Expression in Murine Choroid Plexus via an Oestrogen Receptor Dependent Pathway,” Cellular and Molecular Neurobiology 29, no. 4 (2009): 475–483. 19130215 10.1007/s10571-008-9339-1 PMC11506150

[13] I. Gonçalves , C. H. Alves , T. Quintela , et al., “Transthyretin Is Up‐Regulated by Sex Hormones in Mice Liver,” Molecular and Cellular Biochemistry 317 (2008): 137–142. 18568387 10.1007/s11010-008-9841-2

[14] S. L. Klein and K. L. Flanagan , “Sex Differences in Immune Responses,” Nature Reviews. Immunology 16, no. 10 (2016): 626–638. 10.1038/nri.2016.90 27546235

[15] S. E. Dunn , W. A. Perry , and S. L. Klein , “Mechanisms and Consequences of Sex Differences in Immune Responses,” Nature Reviews. Nephrology 20, no. 1 (2024): 37–55. 37993681 10.1038/s41581-023-00787-w

[16] US Food and Drug Administration , “EPLONTERSEN. Highlights of Prescribing Information,” 2025 https://www.accessdata.fda.gov/drugsatfda_docs/label/2025/217388Orig1s004lbl.pdf.

[17] Canadian Agency for Drugs and Technologies in Health , “Eplontersen Approval,” 2024 https://www.cda‐amc.ca/sites/default/files/DRR/2024/SR0826REC‐Wainua_FINAL.pdf.

[18] European Medicines Agency , “Wainzua (Eplontersen) an Overview of Wainzua and Why it is Authorised in the EU,” 2025 https://www.ema.europa.eu/en/documents/overview/wainzua‐epar‐medicine‐overview_en.pdf.

[19] T. Coelho , W. Marques, Jr. , N. R. Dasgupta , et al., “Eplontersen for Hereditary Transthyretin Amyloidosis With Polyneuropathy,” Journal of the American Medical Association 330, no. 15 (2023): 1448–1458. 37768671 10.1001/jama.2023.18688 PMC10540057

[20] T. Coelho , Y. Ando , M. D. Benson , et al., “Design and Rationale of the Global Phase 3 NEURO‐TTRansform Study of Antisense Oligonucleotide AKCEA‐TTR‐L_Rx_ (ION‐682884‐CS3) in Hereditary Transthyretin‐Mediated Amyloid Polyneuropathy,” Neurology and Therapy 10, no. 1 (2021): 375–389. 33638113 10.1007/s40120-021-00235-6 PMC8140170

[21] T. Coelho , M. Waddington Cruz , C. C. Chao , et al., “Characteristics of Patients With Hereditary Transthyretin Amyloidosis‐Polyneuropathy (ATTRv‐PN) in NEURO‐TTRansform, an Open‐Label Phase 3 Study of Eplontersen,” Neurology and Therapy 12, no. 1 (2023): 267–287. 36525140 10.1007/s40120-022-00414-z PMC9837340

[22] I. Conceicao , J. L. Berk , M. Weiler , et al., “Switching From Inotersen to Eplontersen in Patients With Hereditary Transthyretin‐Mediated Amyloidosis With Polyneuropathy: Analysis From NEURO‐TTRansform,” Journal of Neurology 271, no. 10 (2024): 6655–6666. 39138650 10.1007/s00415-024-12616-6 PMC11447117

[23] A. Aimo , D. Tomasoni , A. Porcari , et al., “Left Ventricular Wall Thickness and Severity of Cardiac Disease in Women and Men With Transthyretin Amyloidosis,” European Journal of Heart Failure 25, no. 4 (2023): 510–514. 36919654 10.1002/ejhf.2824

[24] O. B. Suhr , I. M. Conceição , O. N. Karayal , F. S. Mandel , P. E. Huertas , and B. G. Ericzon , “Post Hoc Analysis of Nutritional Status in Patients With Transthyretin Familial Amyloid Polyneuropathy: Impact of Tafamidis,” Neurology and Therapy 3, no. 2 (2014): 101–112. 26000226 10.1007/s40120-014-0023-8 PMC4386428

[25] J. Wixner , I. Conceição , J. L. Berk , et al., “Neuropathy Impairment and Nutritional Status With Eplontersen in Patients With Hereditary Transthyretin‐Mediated Amyloidosis,” Amyloid 33, no. 1 (2026): 85–89. 41147681 10.1080/13506129.2025.2577361

[26] Y. Ando , T. Coelho , J. L. Berk , et al., “Guideline of Transthyretin‐Related Hereditary Amyloidosis for Clinicians,” Orphanet Journal of Rare Diseases 8 (2013): 31. 23425518 10.1186/1750-1172-8-31 PMC3584981

[27] A. G. Caponetti , C. Rapezzi , C. Gagliardi , et al., “Sex‐Related Risk of Cardiac Involvement in Hereditary Transthyretin Amyloidosis: Insights From THAOS,” JACC Heart Fail 9, no. 10 (2021): 736–746. 34391735 10.1016/j.jchf.2021.05.005

[28] D. Cherepanov , M. Palta , D. G. Fryback , and S. A. Robert , “Gender Differences in Health‐Related Quality‐Of‐Life Are Partly Explained by Sociodemographic and Socioeconomic Variation Between Adult Men and Women in the US: Evidence From Four US Nationally Representative Data Sets,” Quality of Life Research 19, no. 8 (2010): 1115–1124. 20496168 10.1007/s11136-010-9673-x PMC2940034

[29] J. A. Louzado , M. Lopes Cortes , M. Galvão Oliveira , et al., “Gender Differences in the Quality of Life of Formal Workers,” International Journal of Environmental Research and Public Health 18, no. 11 (2021): 5951. 34206069 10.3390/ijerph18115951 PMC8199320

[30] H. Ko , Y. H. Park , B. Cho , et al., “Gender Differences in Health Status, Quality of Life, and Community Service Needs of Older Adults Living Alone,” Archives of Gerontology and Geriatrics 83 (2019): 239–245. 31102926 10.1016/j.archger.2019.05.009

[31] M. C. Babcock , L. E. DuBose , T. L. Witten , et al., “Oxidative Stress and Inflammation Are Associated With Age‐Related Endothelial Dysfunction in Men With Low Testosterone,” Journal of Clinical Endocrinology and Metabolism 107, no. 2 (2022): e500–e514. 34597384 10.1210/clinem/dgab715 PMC8764347

[32] C. A. Obi , W. C. Mostertz , J. M. Griffin , and D. P. Judge , “ATTR Epidemiology, Genetics, and Prognostic Factors,” Methodist DeBakey Cardiovascular Journal 18, no. 2 (2022): 17–26. 10.14797/mdcvj.1066 PMC8932385 35414855

