# Supplementary material for: Eplontersen for Hereditary Transthyretin Amyloidosis With Polyneuropathy: An Exploratory Analysis of Treatment Effect in Male and Female Patients
Source: Muscle Nerve. 2026 Apr 6;73(6):1118–27. doi: 10.1002/mus.70230 (PMC13138353; doi:10.1002/mus.70230)
Supplement: Supplementary file 1 — Table S1. Polyneuropathy Disability disease staging. Table S2. Treatment effect of eplontersen vs. placebo at Week 65/66 for male vs. female patients. Figure S1. Percentage change from baseline to Week 65 in serum TTR levels in male and female patients with CM. Figure S2. Change from baseline to Week 66 in mNIS+7 composite score and Norfolk QoL‐DN total score in male and female patients with CM. [file MUS-73-1118-s001.docx]

# Supplementary Materials for *Muscle & Nerve*:

Eplontersen for Hereditary Transthyretin Amyloidosis With Polyneuropathy: An Exploratory Analysis of Treatment Effect in Male and Female Patients

**Márcia Waddington Cruz, MD, PhD,^1,2^ John L. Berk, MD,^3^ Yeşim Parman, MD,^4^
Morie Gertz, MD,^5^ Sami Khella, MD, FCPP,^6^ Markus Weiler, MD,^7^ T. Jesse Kwoh, PhD,^8^ Maksym Pola, MD, PhD,^9^ Barry Reicher, MD,^10^ Jonatan Nåtman, MSc,^11^ Noel R. Dasgupta, MD,^12^ Jonas Wixner, MD, PhD^13^**

^1^CEPARM, Federal University of Rio de Janeiro, Rio de Janeiro, Brazil; ^2^Amyloidosis Center. Américas-Samaritano-Vitória Hospital Complex, Barra da Tijuca, Rio de Janeiro, Brazil. ^3^Boston University School of Medicine, Boston, Massachusetts, USA; ^4^İstanbul Üniversitesi - Istanbul Tıp Fakültesi, Istanbul, Turkey; ^5^Department of Hematology, Mayo Clinic, Rochester, Minnesota, USA; ^6^University of Pennsylvania School of Medicine, Philadelphia, Pennsylvania, USA; ^7^Amyloidosis Center and Department of Neurology, Heidelberg University Hospital, Heidelberg, Germany; ^8^Clinical Development, Ionis Pharmaceuticals, Inc., Carlsbad, California, USA; ^9^Late Cardiovascular, Renal, Metabolism, BioPharmaceuticals R&D, AstraZeneca, Gothenburg, Sweden; ^10^BioPharmaceuticals R&D, AstraZeneca, Gaithersburg, Maryland, USA; ^11^BioPharmaceuticals Business Unit, AstraZeneca, Gothenburg, Sweden; ^12^Indiana University School of Medicine, Indianapolis, Indiana, USA; ^13^Amyloidosis Centre, Department of Public Health and Clinical Medicine, Umeå University, Umeå, Sweden.

Running header: Eplontersen for male and female patients with ATTR-PN

Correspondence to: Dr Márcia Waddington Cruz, CEPARM, Federal University of Rio de Janeiro, Rio de Janeiro, Brazil.
Email: [mwaddingtoncruz@gmail.com](mailto:mwaddingtoncruz@gmail.com); Telephone: +55 39382112

TABLE S1. PND disease staging.

| **PND stage** | **Symptoms** |
| --- | --- |
| 0 | No impairment |
| I | Sensory disturbances but preserved walking capacity |
| II | Impaired walking capability but ability to walk without a stick or crutches |
| IIIa | Walking only with the help of one stick or crutch |
| IIIb | Walking with the help of two sticks or crutches |
| IV | Confined to a wheelchair or bedridden |

Abbreviation: PND, Polyneuropathy Disability.

TABLE S2. Treatment effect of eplontersen vs. placebo at Week 65/66 for male vs. female patients.

| **Endpoint** | **Male** | | **Female** | | ***p*  (male vs. female)** |
| --- | --- | --- | --- | --- | --- |
|  | **Eplontersen** | **Historical Placebo** | **Eplontersen** | **Historical Placebo** |  |
| **Serum TTR levels, Week 65** | | | | | |
| *n* | 93 | 36 | 42 | 15 |  |
| LSM (SE) [95% CI] | −79.69 (1.84)  [−83.32 to −76.06] | −6.27 (2.93)  [−12.05 to −0.50] | −85.87 (2.68)  [−91.16 to −80.58] | −16.55 (4.43)  [−25.29 to −7.80] | – |
| LSMD (SE) [95% CI] | −73.41 (3.54)  [−80.40 to −66.43] | | −69.32 (5.18)  [−79.54 to −59.09] | | 0.506 |
| **mNIS+7 composite score, Week 66** | | | | | |
| *n* | 88 | 37 | 40 | 15 |  |
| LSM (SE) [95% CI] | 1.3 (1.9)  [−2.3 to 5.0] | 24.6 (2.9)  [18.9 to 30.3] | −3.7 (2.8)  [−9.2 to 1.8] | 24.3 (4.5)  [15.4 to 33.3] | – |
| LSMD (SE) [95% CI] | −23.3 (3.4)  [−30.0 to −16.5] | | −28.0 (5.3)  [−38.5 to −17.5] | | 0.450 |
| **Norfolk QoL-DN total score, Week 66** | | | | | |
| *n* | 91 | 37 | 37 | 15 |  |
| LSM (SE) [95% CI] | −5.9 (1.9)  [−9.6 to −2.3] | 12.7 (2.9)  [6.9 to 18.4] | −10.2 (2.9)  [−16.0 to −4.4] | 9.7 (4.5)  [0.8 to 18.7] | – |
| LSMD (SE) [95% CI] | −18.6 (3.5)  [−25.4 to −11.8] | | −19.9 (5.4)  [−30.6 to −9.3] | | 0.840 |
| **NSC total score, Week 66** | | | | | |
| *n* | 91 | 37 | 41 | 15 |  |
| LSM (SE) [95% CI] | 0.1 (0.8)  [−1.5 to 1.6] | 8.6 (1.2)  [6.2 to 11.0] | −2.5 (1.2)  [−4.8 to −0.2] | 5.9 (1.9)  [2.2 to 9.6] | – |
| LSMD (SE) [95% CI] | −8.5 (1.4)  [−11.4 to −5.7] | | −8.4 (2.2)  [−12.8 to −4.0] | | 0.969 |
| **SF-36 PCS, Week 65** | | | | | |
| *n* | 94 | 35 | 42 | 15 |  |
| LSM (SE) [95% CI] | 0.8 (0.7)  [−0.5 to 2.1] | −3.7 (1.1)  [−5.9 to −1.6] | 2.6 (1.0)  [0.7 to 4.6] | −4.8 (1.6)  [−8.1 to −1.6] | – |
| LSMD (SE) [95% CI] | 4.5 (1.3)  [2.0 to 7.0] | | 7.4 (1.9)  [3.7 to 11.2] | | 0.201 |
| **mBMI, Week 65** | | | | | |
| *n* | 92 | 34 | 38 | 15 |  |
| LSM (SE) [95% CI] | −5.4 (8.5)  [−22.1 to 11.2] | −74.9 (13.6)  [−101.6 to −48.1] | −2.2 (13.2)  [−28.2 to 23.8] | −96.5 (20.4)  [−136.8 to −56.2] | – |
| LSMD (SE) [95% CI] | 69.4 (16.0)  [37.9 to 101.0] | | 94.3 (24.3)  [46.3 to 142.3] | | 0.394 |

Abbreviations: CI, confidence interval; LSM, least squares mean; LSMD, least squares mean difference; mBMI, modified body mass index; mNIS+7, modified Neuropathy Impairment Score +7; NCS, Neuropathy Symptom and Change; Norfolk QoL-DN, Norfolk Quality of Life-Diabetic Neuropathy; PCS, Physical Component Summary; SE, standard error; SF-36, Short Form-36 questionnaire; TTR, transthyretin.

FIGURE S1. Percentage change from baseline to Week 65 in serum TTR levels in male and female patients with CM.


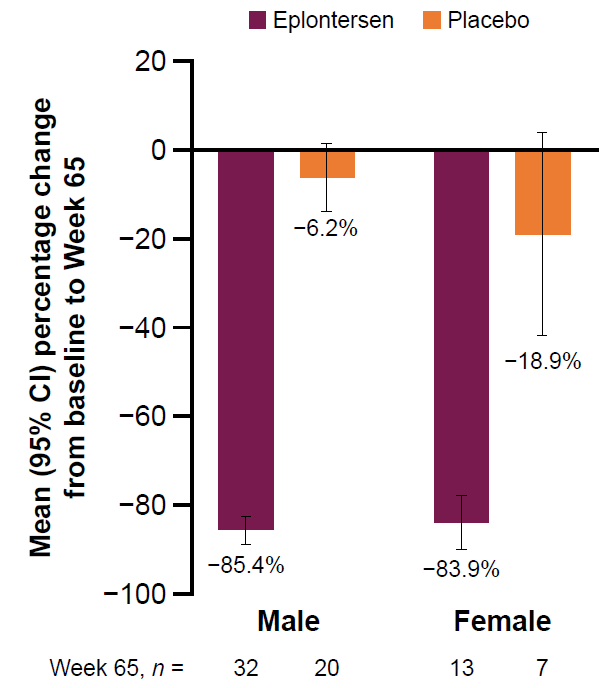


CI, confidence interval; CM, cardiomyopathy; TTR, transthyretin.

FIGURE S2. Change from baseline to Week 66 in mNIS+7 composite score and Norfolk QoL-DN total score in male and female patients with CM.


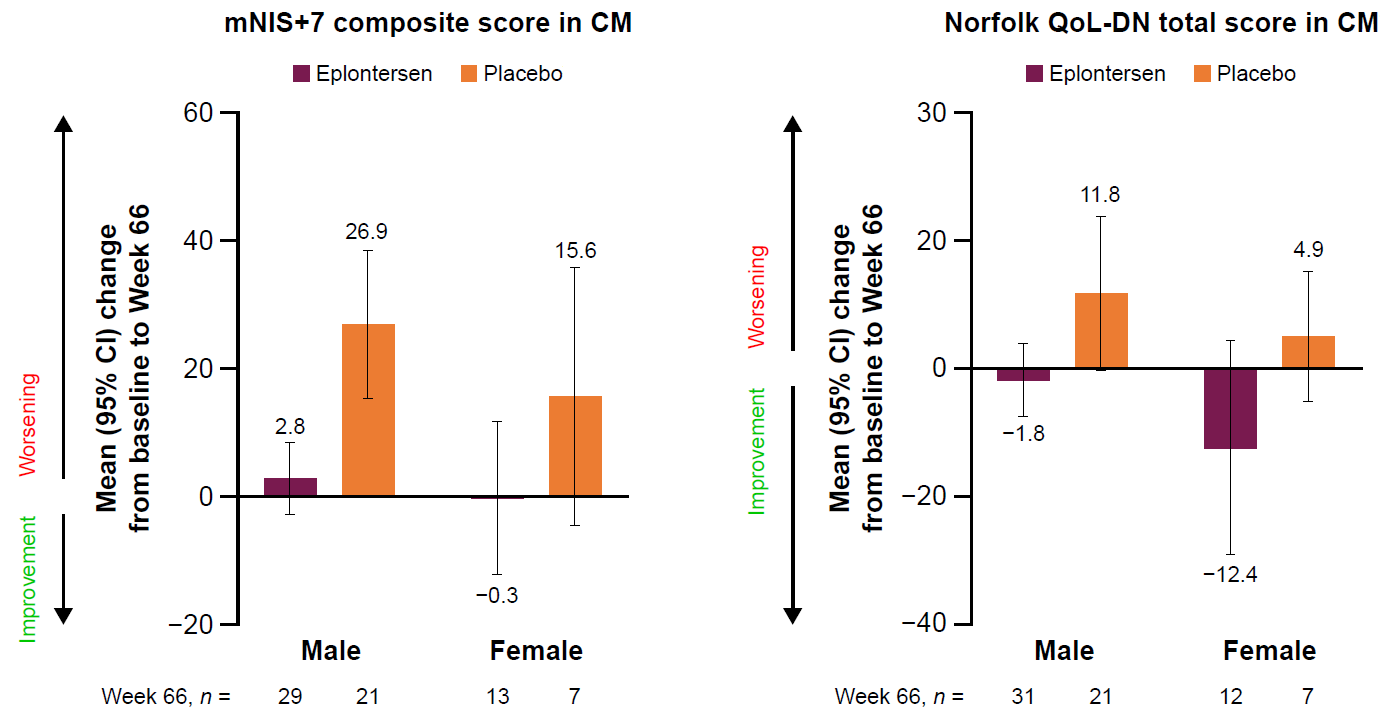


CI, confidence interval; CM, cardiomyopathy; mNIS+7, modified Neuropathy Impairment Score +7; Norfolk QoL-DN, Norfolk Quality of Life-Diabetic Neuropathy.
